# Analysis of conglutin seed storage proteins across lupin species using transcriptomic, protein and comparative genomic approaches

**DOI:** 10.1186/s12870-015-0485-6

**Published:** 2015-04-19

**Authors:** Rhonda C Foley, Jose C Jimenez-Lopez, Lars G Kamphuis, James K Hane, Su Melser, Karam B Singh

**Affiliations:** CSIRO Agriculture Flagship, Centre for Environment and Life Sciences, Floreat, WA Australia; The UWA Institute of Agriculture, University of Western Australia, Crawley, WA Australia; Current address: Curtin Institute for Computation and Centre for Crop & Disease Management, Department of Environment & Agriculture, Curtin University, Bentley, Western Australia Australia

**Keywords:** Seed storage, Seed development, Legumes, Seed transcriptome, Lupin varieties

## Abstract

**Background:**

The major proteins in lupin seeds are conglutins that have primary roles in supplying carbon, sulphur and nitrogen and energy for the germinating seedling. They fall into four families; α, β, γ and δ. Interest in these conglutins is growing as family members have been shown to have beneficial nutritional and pharmaceutical properties.

**Results:**

An in-depth transcriptome and draft genome from the narrow-leafed lupin (NLL; *Lupinus angustifolius*) variety, Tanjil, were examined and 16 conglutin genes were identified. Using RNAseq data sets, the structure and expression of these 16 conglutin genes were analysed across eight lupin varieties from five lupin species. Phylogenic analysis suggest that the α and γ conglutins diverged prior to lupin speciation while β and δ members diverged both prior and after speciation. A comparison of the expression of the 16 conglutin genes was performed, and in general the conglutin genes showed similar levels of RNA expression among varieties within species, but quite distinct expression patterns between lupin species. Antibodies were generated against the specific conglutin families and immunoblot analyses were used to compare the levels of conglutin proteins in various tissues and during different stages of seed development in NLL, Tanjil, confirming the expression in the seed. This analysis showed that the conglutins were expressed highly at the mature seed stage, in all lupin species, and a range of polypeptide sizes were observed for each conglutin family.

**Conclusions:**

This study has provided substantial information on the complexity of the four conglutin families in a range of lupin species in terms of their gene structure, phylogenetic relationships as well as their relative RNA and protein abundance during seed development. The results demonstrate that the majority of the heterogeneity of conglutin polypeptides is likely to arise from post-translational modification from a limited number of precursor polypeptides rather than a large number of different genes. Overall, the results demonstrate a high degree of plasticity for conglutin expression during seed development in different lupin species.

**Electronic supplementary material:**

The online version of this article (doi:10.1186/s12870-015-0485-6) contains supplementary material, which is available to authorized users.

## Background

Legumes form a very important group of crop plants for both animal and human consumption [1]. The genus *Lupinus* comprises between 200 and 600 species, the majority of which have not been domesticated. *Lupinus* are part of the genistoid clade which is quite distinct from other legume sister clades and are the least exploited group of legumes. Interest in lupins is growing as they have a number of attractive nutritional attributes relating to their high protein and dietary fibre contents and negligible starch. Recently, studies have also associated lupins with playing positive roles in health in areas such as combating obesity, diabetes and cardiovascular disease [2-4]. For example, studies have demonstrated that γ conglutin has an ability to reduce glycaemia to levels comparable to those obtained with metformin, a widely used hypoglycaemic drug [4]. Lupins are also valuable in sustainable agricultural systems through their ability to fix nitrogen, they also contribute high organic matter to the soil and are effective in breaking disease cycles for other crops such as cereals [5]. Cultivation of lupins has occurred for thousands of years, and extensive boiling and steeping were required to rid the grain of their bitter and poisonous alkaloids. Modern lupins have been bred to have reduced alkaloid levels and are referred to as sweet lupins [6,7].

The main seed storage proteins in lupins, referred to as conglutins, have been classified into four families; α, β, γ and δ conglutins. α conglutins have been shown to be degraded proteolytically at germination, confirming their role as a classical seed storage protein [8]. β conglutins are abundant seed proteins as verified by quantitating protein [9] and transcript expression [10]. The bean β conglutin homolog, phaseolin, degrades during germination in a phosphorylation-dependent manner [11]. As well as having a seed storage protein function, metabolism of β conglutins also acts to release lectins during germination [12]. β conglutins have also been shown to be potential allergens [10]. γ conglutins may not fall into the classical seed storage category as the protein is not degraded like traditional seed storage proteins during germination, and γ conglutins may also possess lectin-like activity [13]. Although γ conglutins share structural similarities to xyloglucan-specific endo-beta-1,4-glucanase inhibitors, they lack the functional activity of glucanase inhibitors [14]. δ conglutins have been the least studied conglutin family. They are small proteins that are localised in the vacuole [15], and have low-digestibility properties [16].

In this study we analysed the conglutins in a number of lupin species. *L. angustifolius,* (narrow-leafed lupin, NLL) is currently the most extensively cultivated lupin crop and is grown predominantly in South-Western Australia. Three NLL varieties were used in this study; P27255 is a wild variety available prior to domestication of NLL within Australia; Unicrop was one of the first domesticated Australian NLL varieties (released in 1973) as a sweet, non-shattering pod variety, and Tanjil is a more recent variety (released in 1998). Tanjil has resistance to the fungal pathogen, anthracnose (*Colletotrichum spp*.) and has emerged as the reference NLL/lupin variety for which a number of genomic resources have/are being developed [17-23].

*L. albus* (white lupin), is the lupin of preference in Europe and much of the nutritional work has been done on this species [3,9,12,14,20]. The two varieties of *L. albus* used in this study were the Kiev mutant, a Ukrainian bred variety, and Andromeda, a variety with intermediate resistance to anthracnose. Similarly to *L. angustifolus* and *L. albus*, *L. luteus* is an ‘Old World’-smooth seeded species*. L. luteus* (yellow lupin) originates from the Mediterranean coastal regions and was domesticated as a grain legume in Europe by selecting for low alkaloid content, non-shattering pods and soft seeds. The *L. luteus* variety, Pootalong, used in this study is well adapted to acidic soils and the species is favoured by the aquaculture industry because of the high protein content. A *L. luteus’* transcriptome has been generated using 454-expressed sequence tag libraries, and comparative studies using model legume species have identified a comprehensive set of molecular markers for yellow lupin [24]. In addition, a comprehensive seed-protein catalog has been developed for *L. luteus* [25]. *L. mutabilis* (Andean or Pearl Lupin) is known as a ‘New World’ lupin as it originated from South America. *L. mutabilis* has high oil, low alkaloid levels and high protein content. The *L. mutabilis* variety, ID13 was chosen for this study. *L. cosentinii* Guss (sandplain blue lupin) is referred to as an ‘Old World’ – rough seeded lupin and originated from North Africa. It grows prolifically in coastal South Western Australia. Breeding of this variety to produce a sweeter version developed the variety, Erregulla used in this study. However Erregulla, was highly susceptible to aphids and anthracnose and therefore was not released commercially.

This work expands on the study of NLL lupin conglutins reported by Foley et al. [10], and adds new information on their gene structure and RNA and protein expression patterns both in NLL and other lupin species. Previously we reported the identification of sixteen individual seed storage protein genes and investigation of their expression in the species NLL cv Tanjil using EST sequencing [10]. In this study, analysis of an in-depth Tanjil transcriptomic and draft genome sequence [23] did not reveal any additional conglutins in Tanjil. The 16 Tanjil conglutin genes were used to identify homologous genes in seven other lupin varieties from five different lupin species using RNAseq data, which was also used to compare their respective RNA expression levels in seeds. Antibodies designed specifically against each conglutin family were used to analyse the protein levels of these four conglutin protein families in different tissues and at specific stages of seed development. In addition, the levels of these proteins among lupin varieties were compared.

## Results and discussion

### Identification of conglutins sequence reads in lupin species

Transcriptomic studies using RNAseq data are valuable in identifying homologous sequences and RNA expression levels. In NLL a total of three α conglutins, seven β conglutins, two γ conglutins and four δ conglutins were previously identified [10]. A draft genome survey assembly and comprehensive transcriptome assembly for Tanjil [23] were searched to confirm the sequences of these conglutins and to potentially identify any additional conglutins with sequence similarity (<1^e-5^). This analysis confirmed the sequences of all α, γ and δ conglutins identified previously [10] and did not identify any additional conglutins in the NLL cv. Tanjil. In the case of the β conglutins, sequences for BETA1, 2, 5 and 7 were identified, whereas BETA3, 4 and 6 appeared to be mis-assembled in the survey genome assembly and formed contigs with alternate isoforms in the transcriptome assembly, which is most likely due to their high sequence similarity (85-99 % identity) [10]. As the genome survey only comprises 521 Mb of an estimated 924 Mb of genome sequence there remains the possibility that some additional conglutins have not been identified in the genome sequence. However, given that the Tanjil transcriptome, which contained a wide range of different developmental seed stages, and the additional seed transcriptome sequencing for Tanjil presented herein did not show any other transcripts with homology to the conglutins identified previously [10], it would appear unlikely that other conglutins are present in NLL cv. Tanjil.

The conglutins from NLL cv. Tanjil were subsequently used as reference sequences to assemble homologous sequences from transcriptome datasets generated for NLL cv. Unicrop and wild accession P27255, as well as other species in the genus *Lupinus*. This allowed consensus sequences for the eight lupin varieties/species to be retrieved by aligning the RNA seq data for each variety to the 16 Tanjil conglutin sequences. In the case of Tanjil, the extracted 16 conglutin consensus sequences from the RNAseq dataset were compared to the published NLL Tanjil sequences and these aligned very closely (99.6% -100% identity). In some instances stretches of unknown nucleotides were generated from the consensus extraction for other species, likely because of stretches of weaker alignment to Tanjil conglutins.

### Phylogenetic relationships among conglutins from different lupin species

**Figure 1:**
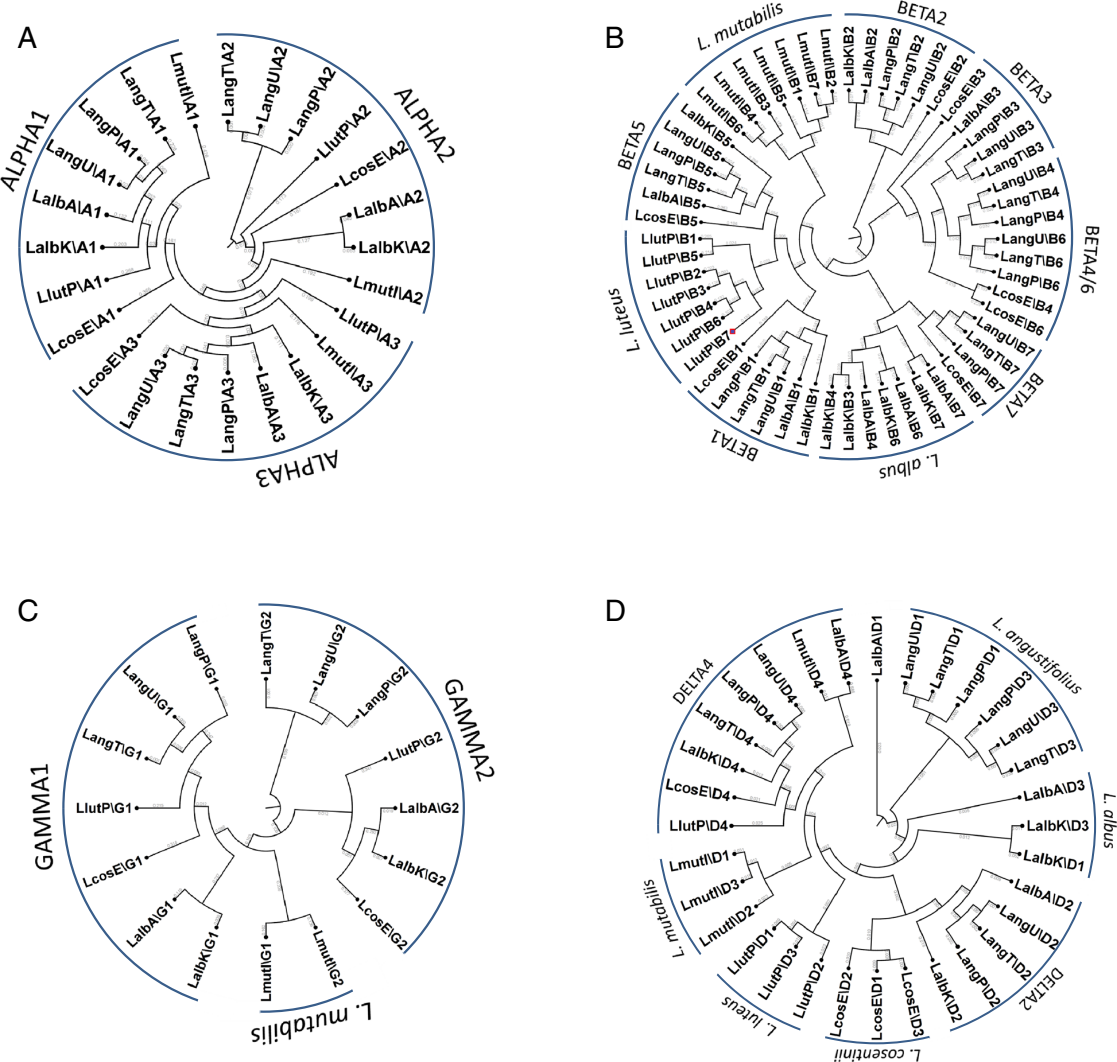
Alignment using the Kmer program of **A)** ALPHA (1-3), **B)** BETA (1-7), **C)** GAMMA (1-2) and **D)** DELTA (1-4) DNA sequences extracted from RNAseq data sets from *L. albus* Andromeda (LalbA*), L. albus* Kiev (LalbK*), L. angustifolus* P27255 (LangP), *L. angustifolius* Tanjil (LangT), *L. angustifolius* Unicrop (LangU), *L. cosentinii* Erregulla (LcosE), *L. luteus* Pootalong (LlutP) and *L. mutabilis* ID13 (LmutI). The branch numbers and relative distance of the tree represent the amount of evolutionary divergence between two nodes in the tree, which was calculated using a k-mer based method in the CLC Genomics WorkBench v7.5.1

Each family of conglutin sequences generated from the eight varieties were aligned using the K-mer program within the CLC Genomics Workbench as shown in Figure 1. With respect to the α family, each lupin variety had three α sequences that were assembled against the Tanjil reference genes. In all instances, the α conglutins were able to be subdivided into three subgroups; ALPHA1, ALPHA2 or ALPHA3 (Figure 1A). This is in agreement with our previous observations which showed that the α conglutin, also known as 11S globulin subgroups, have duplicated and diverged prior to speciation in soybean, pea, peanut, medicago and NLL [10].

In contrast to the α conglutins, the β conglutin sequence relationships were not as clear cut (Figure 1B). While there are some β conglutins that appear to have diverged prior to lupin speciation (for example, BETA1 and BETA2), other examples suggest that the β conglutins are most closely aligned with each other within a species (e.g. *L. mutabilis, L. albus* and *L. luteus*), indicating that they diverged after speciation. Whether these different β sequences are distinct genes or alleles remain to be determined. When comparing β conglutin-like protein (or 7S globulins) relationships in legumes such as soybean, pea, peanut, medicago and NLL, gene duplication in each legume species appeared to have occurred after speciation.

As observed with the α conglutins the γ conglutins fall into two classes among nearly all the lupin varieties examined; GAMMA1 and GAMMA2, with the closest homology found within species (e.g. *L. angustifolius* and *L. albus*) (Figure 1C). Interestingly, *L. mutabilis* GAMMA1 and GAMMA2 did not fall neatly into the two classes with most sequence homology found with each other rather than respective members of the GAMMA1 and GAMMA2 orthologs.

The phylogenic relationships among δ conglutins is more complex compared to α and γ conglutins (Figure 1D). DELTA4 appeared to have arisen prior to speciation, while for *L. cosentinii, L. luteus* and *L. mutabilis* the other three δ conglutins have most homology to the δ conglutins within their own species. For *L. albus* and *L. angustifolius* DELTA2 conglutins show strong sequence similarity between the two species, whereas DELTA1 and DELTA3 conglutins show stronger homology to each other, within the respective species. The phylogenic tree thus illustrates that varieties within the same species are most similar to each other and among the lupin species examined *L. mutabilis* appears most phylogenetically distinct. These results are in agreement with previous findings using ITS sequencing and mass spectrometric fingerprints of seed proteins for defining *Lupinus* spp. relationships [26,27].

### RNAseq vs ESTseq: Analysis of conglutin RNA expression in narrow-leafed lupin cv Tanjil

Gene expression has been studied through a variety of methods including northern blots, quantitative reverse transcriptase-PCR, microarray analyses and most recently, RNAseq. RNAseq has the advantage over previous techniques in that it is more sensitive, is not limited to a set of predetermined probes and also detects transcriptional modification such as alternative splicing and gene fusion. A standard method of describing transcript expression in RNAseq datasets is to express the number of sequence reads as *r*eads *p*er *k*ilobase per *m*illion reads (RPKM), [28]. However, there have been concerns that the accuracy of the calculation of RPKM used in RNAseq may be influenced by gene length, GC content and dinucleotide frequencies [29].

**Figure 2:**
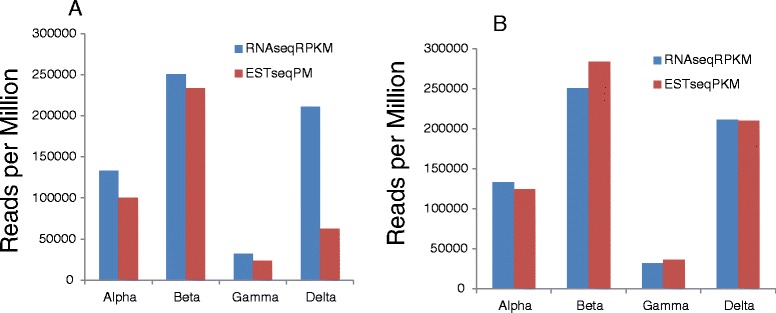
Transcription expression of the four conglutin families from *L. angustifolius*, Tanjil. The RNAseq data was compared to ESTseq data from the same stage of seed development. The EST sequences are represented as **A)** Reads per million (RPM) and **B)** reads per kilobase per million (RPKM).

RNAseq analysis was used to study the expression of the conglutins in NLL cv. Tanjil. The RPKM for the data retrieved for Tanjil was compared to the data obtained from sequencing 3017 clones from a cDNA library, which generated 2381 sequence-readable ESTs [10] from the same stage of seed development and was expressed as *r*eads *p*er *m*illion (RPM) (Figure 2A). The comparison between the two techniques resulted in 32% more reads for α, 7% more reads for β, 36% more reads for γ and over three times more reads for δ using RNAseq compared to ESTseq. This resulted in conglutin genes representing 63% of the total reads using RNAseq compared to 42% using ESTseq [10]. The differences may be due to biological variation, as even though the experiment was replicated as closely as possible, differences may have been introduced unintentionally. For example, plant nutrition and environmental conditions have been shown to greatly affect seed storage protein composition [30,31]. Interestingly, the two data sets have similar expression reads if the EST data is also divided by the gene size (Figure 2B). This suggests that the library construction for the EST data set may be proportionally biased towards larger clones and that the RNAseq datasets generated in this study are a more useful means of comparing conglutin gene expression in and across different lupin species.

### Comparison of conglutin RNA expression among lupin species

**Figure 3:**
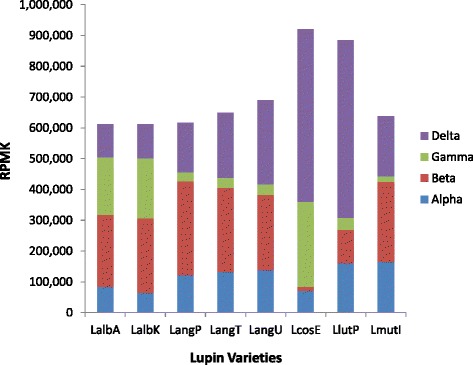
The *r*eads *p*er *k*ilobase per *m*illion reads (RPKM) for each tested lupin species/variety for each family of conglutins (ALPHA, BETA, GAMMA and DELTA). The lupin varieties were *L. albus* Andromeda (LalbA*), L. albus* Kiev (LalbK*), L. angustifolus* P27255 (LangP), *L. angustifolius* Tanjil (LangT), *L. angustifolius* Unicrop (LangU), *L. cosentinii* Erregulla (LcosE), *L. luteus* Pootalong (LlutP) and *L. mutabilis* ID13 (LmutI).

The transcriptomic studies allowed us to directly compare conglutin RNA expression across the five lupin species studied. The RPKM for each family of conglutins in the different lupin species is displayed in Figure 3. As expected, the three varieties of *L. angustifolius* and the two varieties of *L. albus* have similar expression patterns within their respective species. There were some striking differences in conglutin RNA levels between the lupin species. For example, *L. luteus* and *L. cosentinii* have high levels of expression of δ conglutins, with over 60% of conglutin transcripts being δ. In contrast in *L. albus* δ expression was lower and only constituted around 17% of total conglutin transcripts. β conglutins also showed marked differences between lupin species, with β being the most abundant family (>35% of total conglutin transcripts) in *L. angustifolius*, *L. mutabilis* and *L. albus,* while in contrast there were extremely low levels in *L. cosentinii* (less than 2%). While α expression was quite similar across lupin species, γ expression was also quite variable, with high expression in *L. cosentinii* and *L. albus* (~30%) but low expression in the other species (<5%).

**Figure 4:**
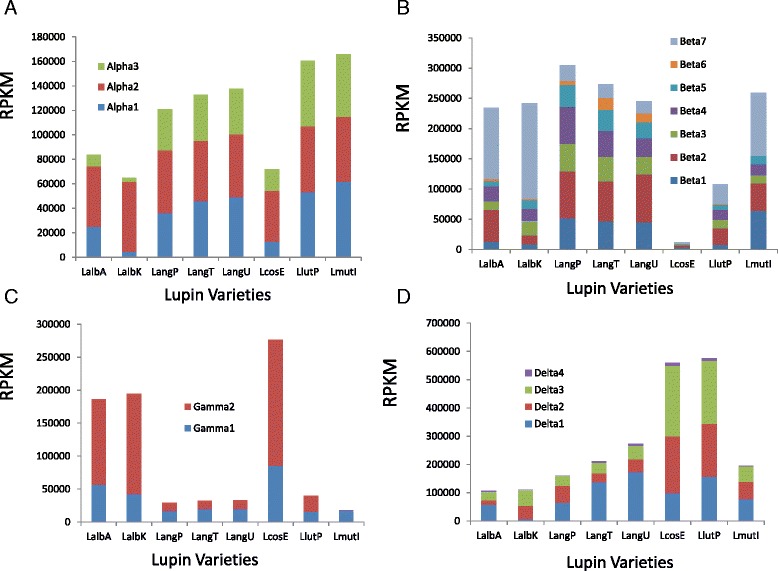
The RPKM for each tested lupin species/variety for each member of the conglutin families. RPKM for conglutin members **A)** Alpha, **B)** Beta, **C)** Gamma and **D)** Delta. The lupin varieties were *L. albus* Andromeda (LalbA*), L. albus* Kiev (LalbK*), L. angustifolus* P27255 (LangP), *L. angustifolius* Tanjil (LangT), *L. angustifolius* Unicrop (LangU), *L. cosentinii* Erregulla (LcosE), *L. luteus* Pootalong (LlutP) and *L. mutabilis* ID13 (LmutI).

The conglutin expression profile can be further analysed to examine the expression of individual conglutin genes. In the case of the α family, ALPHA2 was the major α conglutin expressed in *L. albus* and *L. cosentinii,* but in the other lupin species examined, the different α conglutins were more evenly expressed (Figure 4A). Individual β family members shared substantial differences in their expression levels between lupin species (Figure 4B). For example, BETA7 while being the most abundant in *L. albus* and *L. mutabilis* was one of the least abundant in *L. angustifolius*. Interestingly, a *L. albus* β conglutin sequence has been previously submitted to Genbank as gi:62867685 and this shows greatest identity to BETA7 in *L. albus* Andromeda (98%) and the *L. albus* Kiev mutant (98%). As this is the most abundant β in this species, it was not surprising that this sequence was the first *L. albus* β conglutin sequence reported.

In the case of the γ conglutins, GAMMA1 was the most abundant in *L. mutabilis* and *L. angustifolius* while GAMMA2 was more abundant in the other lupin species (Figure 4C). In the case of the δ conglutins, with the exception of DELTA4, which was expressed at very low levels in all lupin species, the other δ on the whole showed relatively similar proportional expression levels in the different lupin species although DELTA1 expression was much lower in the *L. albus* Kiev mutant than in the *L. albus* Andromeda cultivar (Figure 4D).

### Protein and transcript level comparison in mature seeds

**Figure 5:**
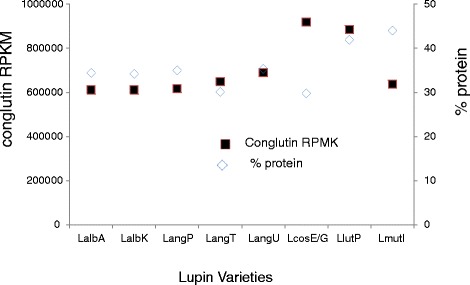
Protein concentrations (% dry weight) of mature seed and total conglutin transcript levels (RPKM) of *L. albus* Andromeda (LalbA)*, L. albus* Kiev (LalbK), *L. angustifolius* P27255 (LangP), *L. angustifolius* Tanjil (LangT), *L. angustifolius* Unicrop (LangU), *L. cosentinii* Guss (LcosG), *L. luteus* Pootalong (LlutP) and *L. mutabilis* ID13 (LmutI).

A number of factors affect protein abundance, including transcription, translation and protein- turnover. To determine if the transcript numbers were correlated with the total protein abundance, the protein concentration in relation to the dry weight of the mature seed was measured. As shown in Figure 5, the cultivars ranged from 30-44% protein content. In the case of *L. albus, L. angustifolius* and *L. luteus*, there was a good correlation between the levels of conglutin transcripts in maturing seeds and the protein abundance in the mature seed, a stage where conglutin transcripts make up a major portion of the total seed transciptome in NLL [10]. However, this correlation was not maintained in *L. cosentinii* and *L.mutabilis* where *L. cosentinii* had the highest level of conglutin transcripts but the lowest percentage protein, while *L. mutabilis* was the opposite. There can be a number of explanations, for example translational regulation, post-translational modification and protein turnover. Moreover, a variety of seed components contribute to the weight of the seed (eg fibre, oil, etc), and these may differentially contribute to the final seed weight in some lupin species. For example, *L. mutabilis* it is known to have relatively high oil levels. Transcript expression of seed storage proteins in *Lotus japonicas* during seed development has been correlated to other genes including oleosins, seed maturation proteins, a late embryogenesis protein and 16 transcription factors [32].

### Conglutin protein analysis during seed development in L. angustifolius, cv Tanjil

**Figure 6:**
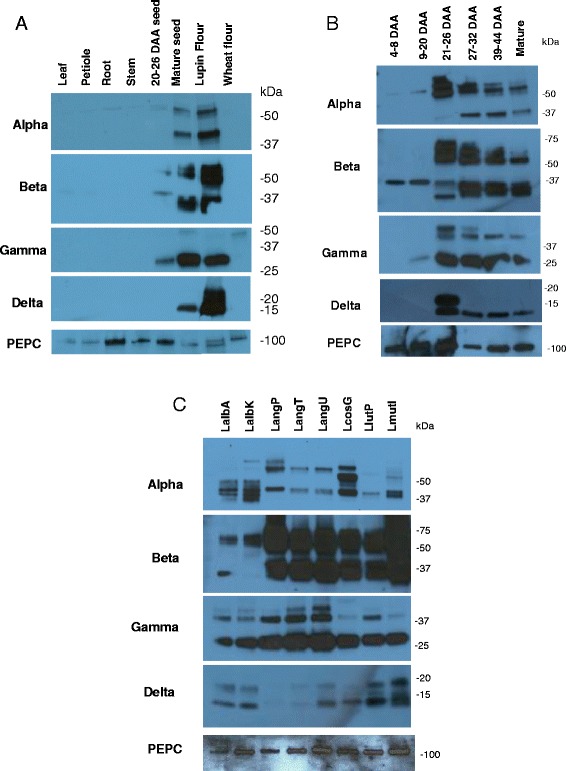
Western blot of 5 ug protein extracts from various lupin samples. The blots were probed using antibodies generated against ALPHA conglutin (expected size 56 kD), BETA conglutin (68-72 kD), GAMMA conglutin (49 kD – uncleaved, 33 kD cleaved), and DELTA conglutin (18 kD– uncleaved, 12 kD cleaved) peptides, and the commercially available PEPC antibody to illustrate similar protein loading. **A)** Protein expression of conglutins in *L. angustifolius* Tanjil and wheat flour. **B)** Protein expression in developmental stages of *L. angustifolius* Tanjil seeds. **C)** Protein expression of conglutin in mature seeds of various lupin species/varietie.

In *L. angustifolius,* cv Tanjil, transcription levels of the conglutin genes was demonstrated to increase during seed development quite dramatically from 4 to 30 *D*ays *A*fter *A*nthesis (DAA) [10]. It was therefore of interest to see if this was reflected in protein abundance, and the relative expression of conglutins in a variety of tissue types. Immunoblot analysis at different stages of seed development and for different tissues types were performed using polyclonal antibodies raised against synthetic peptides derived from each of the conglutin sequences in Tanjil. Comparison of protein expression of the four families of conglutins demonstrate strong tissue specificity with high expression in the seed and no detection in the leaf, petiole, root and stem tissue from two- month old plants (Figure 6A). In addition, protein from commercially available NLL lupin flour and wheat were also tested, demonstrating the proteins remain intact during lupin milling and emphasising the specificity of the antibodies, since no proteins were detected in wheat flour (Figure 6A). Seeds were collected from the pods at six time points that ranged from 4 DAA to mature seed. 20-26 DAA was the developmental stage used for EST sequencing and RNAseq reported in this study. Equal amounts of protein were loaded for each sample and the extracted protein profiles were separated by SDS-PAGE. Figure 6B illustrates that protein bands similar in size to the predicted pro-protein molecular weight range for α (56 kDa) and β (~70 kDa). In the case of BETA conglutins, the expression of pro-protein of predicted size of ~70 kD is higher at the mid-developmental range, and in later time points there is more expression of smaller polypeptides, suggesting post-translation processing is occurring within the maturing seed. GAMMA and DELTA proteins are expressed at higher levels in middle and later developmental seed stages (Figure 6b).

A 2D analysis of the *L. albus* seed proteome found that 37 out of 40 spots fell into the α, β or γ conglutin families, with many of the proteins having a large size range and several endoproteolytically cleaved subunits could be identified [33]. Our results are in agreement with this previous study, where we have observed a range of peptides with different molecular weights in our immunoblot analysis corresponding to the species, *L. angustifolius*. In addition, the heterogeneity of peptides corresponding to α and β conglutins have also been observed from purified conglutins in *L. albus* [34]. As the genomic and transcriptomic analyses have identified a fixed number of conglutin genes, this further supports that the majority of the heterogeneity of conglutin polypeptides is from post-translational modification from a limited number of precursor polypeptides rather than a large number of different genes.

### Analysis of protein expression patterns in different lupin species

The expression profiles of conglutin protein were also analysed in mature seed for the different lupin species/varieties used in the RNAseq studies. Sequence comparisons over the region of the target conglutin peptide used for antibody production, across the lupin species analysed, demonstrated high homology and therefore a high likely hood that the antibodies would recognise conglutins from all tested lupin species (data not shown). Similar levels of total protein were extracted from mature seeds and separated by SBS-PAGE as demonstrated by staining with Coomassie Brilliant blue (data not shown), and using an antibody against phosphoenolpyruvate caroxylase (PEPC). Figure 6C illustrates that differences occur in the protein profile for the different lupin species, although varieties from the same species often showed similar protein profiles, for example, *L. angustifolius* varieties Tanjil and Unicrop. There was no obvious correlation of conglutin transcript expression with final conglutin protein levels. For example, *L. mutabilis* exhibited the highest levels of proteins recognised by the β conglutin antisera but only had average β conglutin transcript expression levels, suggesting that post-transcriptional control is an important factor in conglutin expression in lupin species.

## Conclusion

This study has provided substantial information on the complexity of the four conglutin families in a range of lupin species in terms of their gene structure, phylogenetic relationships and their relative RNA and protein abundance during seed development. These results will provide a good foundation for further research to elucidate the function and nutritional aspects of these seed storage proteins, that in turn will provide the knowledge to try and alter lupin seed protein composition to optimize the health properties of lupins. It was interesting to note that the different lupin species had large variations in conglutin RNA expression. These variations in RNA levels did not appear to have any obvious deleterious effects with regards to seed germination and development. There is evidence to support the plasticity of seed protein expression, for example the ability of sulphur and nitrogen supply to affect seed storage composition [30]. Interestingly, genetically manipulating seed storage proteins in soybean, using RNA interference or mutation results in rebalancing of the proteome thereby maintaining overall wild-type levels of overall seed storage proteins [35,36]. Future work in lupins may involve manipulating seed proteins for optimum nutrition and growth. For example, as β conglutins have been demonstrated to have some allergenic properties, it may be possible to reduce these proteins in favour of γ conglutins that may have higher nutritional benefits.

## Methods

### Plant growth

Lupin plants were grown in a growth cabinet at 22°C/18°C over a 14 h/10 h day/night schedule under 600 Photosynthetically Active Radiation μmol m^-2^ s^-1^ lights. For the transcriptomic comparisons, seeds were collected 20-26 *D*ays *A*fter *A*nthesis (DAA) for, *L. albus* cv Andromeda, *L. albus* cv Kiev mutant, *L. angustifolius* cv P27255, *L. angustifolius* Tanjil, *L. angustifolius* cv Unicrop, *L. cosentinii* cv Erregulla *L. luteus* cv Pootalong,and *L. mutabilis* cv ID13. For the protein level analysis, eight grams of mature seed was supplied to the ChemCentre (www.chemcentre.wa.gov.au) of the above cultivars, except *L. cosentinii* cv Gus was used instead of *L. cosentinii* cv Erregulla, as the Erregulla variety lost its viability to germinate. Determination of protein levels was measured by the amount of nitrogen using thermal conductivity [37,38]. For the immunoblot analysis, seeds were collected from the pods 4 DAA to 53 DAA (mature seed) from *L. angustifolius* Tanjil. Commercially available lupin flour (Irwin Valley. *L. angustifolius*) was also obtained.

### RNA isolation, library preparation, sequencing and quality control

RNA was isolated using the Trizol method [23] as followed: for each of the eight lupin samples approximately 150 mg of maturing seed (20-26 DAA) was ground to a fine powder under a stream of liquid nitrogen. The ground powder was homogenised in 1.5 mL Eppendorf tubes using two-times 500 μL Trizol reagents (Invitrogen, Carlsbad, CA), followed by a 15 min incubation period at room temperature. After centrifugation at 12000 g and 4°C for 10 min the supernatant was mixed with 200 μL chloroform, followed by another centrifugation step at 12000 g and 4°C for 15 min. The upper phase was mixed with 300 μL of high salt precipitation buffer (0.8 M sodium citrate/1.2 M NaCl) and 300 μL of isopropanol and incubated on ice for at least 10 min to selectively precipitate total RNA. Precipitated RNA was centrifuged (10 min 12000 g and 4°C) and washed twice in 75% ethanol. RNA was dissolved in diethylpyrocarbonate treated water. Quality and quantity was assessed by both BioAnalyzer (Agilent, Santa Clara, CA, USA) and Qubit assays (Invitrogen).

One μg of total RNA was used to generate TruSeq RNA libraries (Illumina, San Diego, CA, USA) according to the manufacturer’s recommendations. The eight Lupin TruSeq RNA libraries were pooled evenly and sequenced on a single lane of an Illumina HiSeq1000 on a 2× 100 bp Paired End run.

Raw RNA-Seq data for libraries were trimmed for low quality (<Q30) and Illumina sequencing primer and adaptor sequences (Truseq v2) via Cutadapt 1.1 [39] (overlap 10, times 3, minimum length 25). Reads trimmed to less than 25 bp were discarded and reads with a discarded pair were retained as singleton reads. Transcriptome sequencing and subsequent trimming resulted in quality-controlled RNAseq data ranging from 1.38 to 2.69 Gb per library (Additional file 1: Table S1). The RNAseq and conglutin EST sequences used in this study were submitted to the Sequence Read Archive and dbEST at NCBI under bioproject accession PRJNA271721.

### Data analysis

The conglutin gene sequences identified [10] were used to search the Tanjil survey genome and transcriptome assembly [23], using BLASTN and tBLASTX in CLC Genomics WorkBench 6.0 (CLC Bio, Aarhus, Denmark) to confirm the presence of the conglutins in the genome and transcriptome assembly and to identify if any additional conglutin family members were present.

The Illumina trimmed sequencing reads were sorted into paired and unpaired groups and aligned to the 16 NLL conglutin genes, using CLC Genomics Workbench 6.0. Following the alignment to 16 NLL conglutin reference genes [10] the consensus generated homologous sequence was extracted for each conglutin gene from each variety. The extracted consensus sequences were aligned within each family using K-mer based tree construction (using CLC Genomics Workbench 6). The *r*eads *p*er *k*ilobase per *m*illion reads (RPKM) values were determined for each of the 16 consensus sequences for each lupin variety using the RNAseq datasets and the RNAseq analysis function in CLC Genomics WorkBench 6.0.

### Antibodies

Synthetic peptides for production of the polyclonal serum against α conglutin, δ conglutin and β conglutin were from Mimotopes Pty Ltd, (www.mimotopes.com), and γ conglutin from Agrisera (www.agrisera.com). All peptides were conjugated to keyhole limpet hemocyanin as carrier with a maleimidocaproyl-N-hydroxysuccinimide linker and produced to immunograde purity. The sequences for each peptide synthesized were: α conglutin: (NH2)- CRETYEEPQEQEQG (-CONH2), β conglutin: (NH2-) CVDEGEGNYELVGIR (-CONH2), γ conglutin: (NH2)- CSSTYQAPFCHSTQCSRAN -amidated and δ conglutin: (NH2)- CRAILQQIYESQSEQ (-CONH2). Polyclonal antibodies were raised in rabbits at the following facilities: IMVS Pathology, Veterinary Services Division, University of Adelaide, Australia (β conglutin), Agrisera, (γ conglutin), and the Monoclonal Antibody Facility, Harry Perkins Institute of Medical Research (α conglutin, δ conglutin). Anti-PEPC antiserum was purchased from Agrisera.

### Protein extraction, SDS-PAGE and immunoblot analyses

Seed material (0.5 g) was grounded into a fine powder in a mortar and pestle using liquid nitrogen, and resuspended in a solution containing 100 mM KPO_4_ (pH 7.4), 1 mM EDTA, 1% Triton X-100, 10% glycerol, 1/200 proteinase inhibitor cocktail (Sigma: www.sigmaaldrich.com), 0.5 mM phenylmethanesulfonylfluoride (Sigma) and 1 mM DTT at a concentration of 50% (w/v). After a 15 min spin (14,000 rpm), the supernatant was recovered and measured for protein concentration using the Qubit Protein Assay kit (Invitrogen: Invitrogen.com.au).

Total protein (5 ug per lane) were loaded and separated by SDS-PAGE on a 4-20% acrylamide Mini Protean ®TGX gel (Biorad: www.bio-rad.com) using Mini Protean ®Tetra Cell apparatus (Biorad). To verify protein quantification, one gel was stained with Coomassie Brilliant Blue (Bio-Rad) according to manufacturer’s procedures. The second gel was used for immunoblot analysis.

Proteins were electroblotted onto a PVDF member (Bio-Rad) using a Mini-Trans-Blot Electrophoretic Transfer Cell sysem (Bio-Rad). Western blots were performed using standard techniques [40] with an overnight incubation of 1/1000 dilution of rabbit serum containing polyclonal antibodies followed by TBS washes and a 2 h incubation of goat anti-rabbit IgG horseradish peroxidise conjugate (Bio-Rad). The chemiluminescence signal was detected using Western Lightning Plus-ECL substrate (Perkin Elmer: www.perkinelmer.com) following the manufacturers’ instructions and Xray film (Kodak; www.kodak.com) detection.

### Availability of supporting data

The RNAseq and conglutin EST sequences used in this study have been submitted to the Sequence Read Archive and dbEST at NCBI under bioproject accession PRJNA271721.

## Additional file

Additional file 1: Table S1. RNAseq data information.

## Abbreviations

DAA: Days after anthesis
EST: Expressed sequence tag
LalbA: *L. albus* Andromeda
LalbK: *L. albus* Kiev
LangP: *L. angustifolus* P27255
LangT: *L. angustifolius* Tanjil
LangU: *L. angustifolius* Unicrop
LcosE: *L. cosentinii* Erregulla
LlutP: *L. luteus* Pootalong
LmutI: *L. mutabilis* ID13
NLL: Narrow-leafed lupin
RPKM: Reads per kilobase per million reads
RPM: Reads per million
