# Supplementary material for: Analysis of conglutin seed storage proteins across lupin species using transcriptomic, protein and comparative genomic approaches
Source: BMC Plant Biol. 2015 Apr 19;15:106. doi: 10.1186/s12870-015-0485-6 (PMC4407355; doi:10.1186/s12870-015-0485-6)
Supplement: Additional file 1: Table S1. — RNAseq data information. [file 12870_2015_485_MOESM1_ESM.doc]

Additional file 1: Table S1: RNAseq Data Information

| **Species** | **Cultivar** | **Abbreviation** | **# of reads** | **Average read length** | **Clean RNAseq data** |
| --- | --- | --- | --- | --- | --- |
| *L. albus* | Kiev mutant | LalbK | 25,252,318 | 96.6 | 2.44 Gb |
| *L. albus* | Andromeda | LalbA | 25,667,444 | 96.7 | 2.48 Gb |
| *L. angustifolius* | Unicrop | LangU | 17,048,072 | 96.8 | 1.65 Gb |
| *L. angustifolius* | Tanjil | LangT | 22,707,684 | 96.4 | 2.18 Gb |
| *L. angustifolius* | P27255 | LangP | 22,953,678 | 96.7 | 2.21 Gb |
| *L. cosentinii* | Erregulla | LcosE | 19,968,176 | 96.5 | 1.92 Gb |
| *L. luteus* | Pootalong | LlutP | 14,353,590 | 96.6 | 1.38 Gb |
| *L. mutabilis* | ID13 | LmutI | 27,904,660 | 96.6 | 2.69 Gb |
